# Feasibility Is Not Benefit: Resectable, Irradiable, or Biologically Selected? A Critical Review of the Unresolved Local-Platform Question in Stage III/N2 NSCLC

**DOI:** 10.3390/cancers18203348

**Published:** 2026-10-16

**Authors:** Panteleimonas Skarlos, Ioannis Georgakopoulos, Andromachi Kougioumtzopoulou, Zoi Liakouli, Kiki Theodorou, Anna Zygogianni, Emmanouil Georgiannakis, Vasileios Kouloulias, George Maravelis

**Affiliations:** 1 Department of Radiation Oncology, University Hospital of Larissa, Medical School, University of Thessaly, 41110 Larissa, Greece; p.skarlos@yahoo.gr (P.S.); zliakouli@uth.gr (Z.L.); 2 Radiation Oncology Department, ARETAIEION University Hospital, Medical School, Kapodistrian University of Athens, 11528 Athens, Greece; ioangeo@med.uoa.gr; 3 Department of Clinical Radiation Oncology, University Hospital ATTIKON, Medical School, Kapodistrian University of Athens, 12462 Athens, Greece; andromachi.kou@gmail.com (A.K.); azygogianni@med.uoa.gr (A.Z.); vkouloul@med.uoa.gr (V.K.); 4 Department of Medical Physics, University Hospital of Larissa, Medical School, University of Thessaly, 41110 Larissa, Greece; ktheodor@med.uth.gr; 5 Department of Minimally Invasive Thoracic Surgery, Metropolitan Hospital, 18547 Piraeus, Greece; egeorgiannakis@yahoo.com; 6 Department of Thoracic Surgery, Metropolitan General, 15562 Athens, Greece; 7 Department of Basic and Clinical Sciences, Medical School, University of Nicosia (UNIC), Athens Campus, 16777 Athens, Greece; 8 2nd Department of Radiation Oncology, Metropolitan Hospital, Neo Faliro, 18547 Piraeus, Greece

**Keywords:** N2 NSCLC, stage III NSCLC, perioperative immunotherapy, chemoradiotherapy, durvalumab, resectability, clinical equipoise, randomized trial, TNM ninth edition, treatment selection

## Abstract

Stage III/N2 non-small-cell lung cancer (NSCLC) can often be treated with either surgery-based multimodality therapy or definitive chemoradiotherapy. Historical randomized trials showed that surgery improved local control without demonstrating an overall-survival benefit; neither platform demonstrated superiority, although none was designed to establish equivalence. In the immunotherapy era, both pathways have improved through separate evidence streams: perioperative trials compared immunotherapy with chemotherapy-based surgical controls, and PACIFIC compared durvalumab with placebo after chemoradiotherapy. Neither stream determines which complete modern pathway is better for a patient eligible for both, and cross-trial comparisons are confounded by differences in populations, randomization points, and endpoint definitions. This review distinguishes three questions that are often conflated: whether direct comparative evidence exists, how large any relative treatment effect may be, and whether sufficient clinical equipoise exists to justify randomization. Technical resectability, physiological operability, and irradiability define eligibility for a platform, not comparative benefit, and no current biomarker has been shown to modify the relative effect of the two platforms. Comparative uncertainty is most visible in multistation, bulky, or anatomically complex N2 disease, the most practicable starting point for randomization; superiority of either platform, however, has not been demonstrated at either end of the N2 spectrum. A staged randomized comparison of the complete modern pathways, with prespecified analyses of candidate treatment-effect modifiers, is proposed as a reasoned research strategy.

## Simple Summary

Stage III/N2 non-small-cell lung cancer, in which the cancer has spread to mediastinal (N2) lymph nodes, can often be treated with either a surgery-based approach or definitive chemoradiotherapy. Both strategies have improved substantially with immunotherapy, but they have been studied separately and have not been directly compared using modern treatment. The fact that a tumor can technically be removed does not show that surgery provides better outcomes than chemoradiotherapy, and the fact that it can be irradiated does not show the reverse. This review examines the evidence supporting both approaches, distinguishes the absence of comparative evidence from clinical equipoise, and proposes a staged randomized trial comparing the complete surgery-based and chemoradiotherapy-based pathways in patients eligible for both, to determine whether either provides greater benefit and for which patients.

## 1. Introduction

Stage III/N2 NSCLC lies at the boundary between localized and systemic disease and has long supported two curative strategies: surgery within a multimodality pathway and definitive concurrent chemoradiotherapy (CRT). Some patients are eligible for only one, because R0 resection is not technically achievable, operative risk is unacceptable, or definitive radiotherapy cannot be delivered within protocol constraints. The unresolved population is different: patients in whom both complete pathways are deliverable. Within that dual-eligible population, the comparative question is which complete local–systemic pathway produces the better net outcome. Throughout, the surgery-based pathway denotes resection with neoadjuvant or perioperative systemic therapy, and the chemoradiotherapy-based pathway denotes definitive concurrent platinum-based CRT followed by consolidation immunotherapy (CRT-immunotherapy), the contemporary non-surgical comparator; ‘platform’ denotes the local modality alone, and ‘radiotherapy-based’ is reserved for historical trials whose non-surgical arms did not uniformly use concurrent CRT.

Nodal terminology requires a preliminary note. The ninth edition of the TNM classification subdivides N2 into single-station (N2a) and multistation (N2b) involvement and reclassifies T1N2a disease from stage IIIA to stage IIB [1]. Here, ‘stage III/N2’ denotes the N2 population historically classified as stage III, including the T1N2a subset now classified as stage IIB [2]. Single- and multistation involvement correspond to N2a and N2b where the source data permit. The N2a/N2b subdivision is prognostic: it separates patients by expected survival under the treatments in use when the outcome data were collected, not by relative benefit from either pathway. Whether N2a versus N2b modifies the comparative effect of surgery-based and chemoradiotherapy-based treatment has not been tested, which is why it is treated here as a candidate treatment-effect modifier rather than an allocation rule.

Immune checkpoint inhibition has improved outcomes in both pathways. PACIFIC established consolidation durvalumab after definitive concurrent chemoradiotherapy [3,4,5]. CheckMate 816, KEYNOTE-671, CheckMate 77T, AEGEAN, NADIM/NADIM II, and Neotorch established neoadjuvant or perioperative immunotherapy within surgery-based treatment [6,7,8,9,10,11,12,13,14]. These trials changed practice, but each answered a question within one pathway; none compared the two updated pathways in patients eligible for both (Table 1).

The distinction between feasibility and benefit is therefore central. Technical resectability asks whether an R0 resection can reasonably be achieved. Physiological operability asks whether the patient can tolerate the planned operation. Irradiability asks whether a definitive radiotherapy dose can be delivered within acceptable normal-tissue constraints. These judgments define eligibility for a platform. They do not determine comparative platform suitability: whether one complete pathway produces better survival, function, quality of life, or total treatment burden than the other in the same patient.

Three questions are often conflated. The first is whether direct randomized evidence comparing the two modern pathways exists; it does not, for any part of the dual-eligible N2 population. The second is how large any relative treatment effect may be; this is uncertain, but the uncertainty is informed by prior randomized trials and may differ across subgroups. The third is whether patients and clinicians regard the two pathways as sufficiently balanced to accept randomization (practical equipoise); this varies substantially across the N2 spectrum. Absence of direct evidence does not by itself establish equipoise, and a strong clinical preference does not by itself establish superiority. All three questions differ from formal equivalence or non-inferiority: the historical trials failed to demonstrate superiority of either platform, but none was designed or powered to establish equivalence, and no trial has tested equivalence or non-inferiority between the modern pathways.

**Figure 1:**
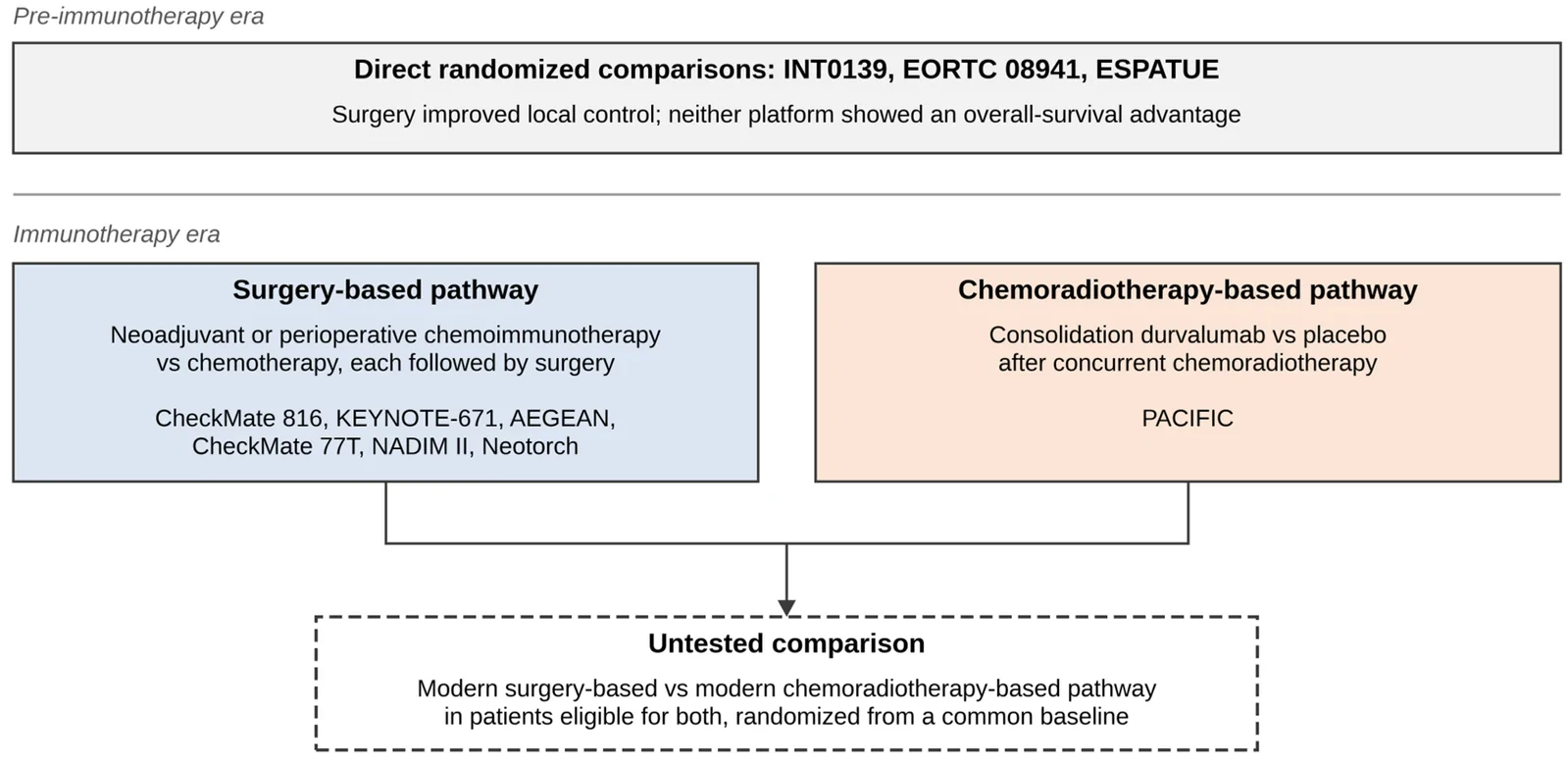
Two parallel evidence streams and the missing comparison in stage III/N2 NSCLC. Historical trials compared surgery-based and radiotherapy-based pathways directly. In the immunotherapy era, each pathway was improved against its own control in separate trials. The comparison that remains untested is between the two complete modern pathways in patients eligible for both. The connector indicates that both modern evidence streams converge on this comparison; it does not indicate a treatment sequence.

This critical narrative review examines the historical randomized evidence, the separate modernization of both pathways, the limitations of cross-trial comparison, the lessons of PIONEER, emerging comparative designs, and the requirements of a contemporary trial (Figure 1). Both pathways are regarded here as legitimate curative options, and current multidisciplinary decisions as appropriately made on the evidence available; the aim is to identify where that evidence ends.

## 2. Materials and Methods

This critical narrative review was based on a literature search of PubMed/MEDLINE, from database inception to July 2026, with an update during revision in October 2026, for publications addressing the comparative role of surgery-based and definitive radiotherapy-based treatment strategies in stage III/N2 NSCLC. Search terms were combined as follows: (‘stage III NSCLC’ OR ‘N2 NSCLC’ OR ‘stage IIIA-N2’ OR ‘resectable NSCLC’) AND (‘resectability’ OR ‘surgery’ OR ‘lobectomy’ OR ‘pneumonectomy’ OR ‘chemoradiotherapy’ OR ‘radiotherapy’) AND (‘durvalumab’ OR ‘perioperative immunotherapy’ OR ‘neoadjuvant immunotherapy’ OR ‘immune checkpoint inhibition’ OR ‘treatment selection’). The search was limited to English-language publications. Reference lists of key publications were screened; consensus statements and guidelines addressing technical resectability, mediastinal staging, and the management of N2 disease were sought specifically; and ongoing comparative trials were identified through trial registries.

Publications were included if they were (i) randomized trials comparing surgery-based with radiotherapy-based treatment in stage III or N2 NSCLC; (ii) randomized trials establishing contemporary systemic therapy within either pathway, with their mature follow-up reports and N2 or nodal-status subgroup analyses; (iii) consensus statements, guidelines, or systematic reviews on resectability, mediastinal staging, or the management of N2 disease; (iv) non-randomized comparisons of the two pathways; (v) studies of anatomical, dosimetric, molecular, or circulating factors relevant to the relative value of the two pathways; or (vi) expert commentaries representing surgical and non-surgical positions, cited as opinion rather than evidence. Excluded were case reports, studies confined to stage I–II disease without N2 involvement, studies confined to metastatic disease unless they informed biomarker biology, and conference abstracts whose data had been published in full; abstracts reporting data not yet published in full were included and are identified as such. Titles, abstracts, and full texts were assessed against these criteria by two authors (P.S. and G.M.), with disagreements resolved by discussion among all authors.

Because the question spans thoracic surgery, radiation oncology, and medical oncology, evidence was selected to represent each discipline: the surgical literature included the perioperative immunotherapy trials, surgical consensus documents, analyses of minimally invasive resection and of pneumonectomy-related mortality, and arguments for a central surgical role [6,7,8,9,10,11,12,13,14,15,16,17,18,19,20], alongside the radiotherapy and chemoradiotherapy literature. In keeping with the narrative design, no PRISMA flow diagram or study counts are reported; selection is therefore subject to the limitations inherent to narrative reviews, which the explicit search terms, eligibility criteria, evidence priorities, and appraisal criteria described here are intended to make transparent.

Priority was given to randomized clinical trials directly comparing surgery-based and radiotherapy-based strategies, trials establishing contemporary systemic treatment within either platform, mature follow-up reports, systematic reviews, recent consensus and guideline documents addressing technical resectability and treatment selection, and prospective or retrospective comparative studies relevant to the clinical question. Particular attention was given to the historical randomized evidence from INT0139 [21], EORTC 08941 [22], and ESPATUE [23,24]; contemporary perioperative immunotherapy trials; and studies establishing definitive concurrent chemoradiotherapy followed by consolidation immunotherapy. Relevant subgroup analyses addressing N2 disease, nodal extent, surgical approach, and treatment-related risk were also considered.

Because the purpose of this review was not to estimate a pooled treatment effect but to critically examine the evidentiary basis for choosing between two complete curative treatment platforms, studies were evaluated according to the clinical question addressed, the population enrolled, the treatment comparator, the timing of randomization, and the endpoints used. Particular attention was paid to differences in patient selection, treatment baseline, and endpoint definitions that limit indirect comparisons between contemporary surgery-based and chemoradiotherapy-based trials.

Evidence concerning technical resectability, physiological operability, radiotherapy feasibility, and normal-tissue dose burden was considered separately from evidence of comparative oncological benefit. Anatomical, physiological, dosimetric, molecular, imaging, and circulating biomarkers were evaluated as potential treatment-effect modifiers when relevant to the relative value of the two treatment platforms, rather than as established criteria for selecting one strategy over the other.

Given the narrative and hypothesis-generating nature of the review and the heterogeneity of the included evidence, no formal meta-analysis or quantitative risk-of-bias assessment was performed. The evidence was synthesized qualitatively, with emphasis on study design, directness of comparison, maturity of follow-up, applicability to contemporary practice, and the distinction between evidence of treatment feasibility and evidence of comparative benefit.

## 3. Results

### 3.1. Historical Randomized Evidence: Neither Platform Demonstrated Superiority

INT0139 directly tested whether surgery after induction concurrent chemoradiotherapy improved outcome in patients with biopsy-proven T1–3 N2 NSCLC considered operable and potentially resectable. Patients were randomized at registration to concurrent cisplatin–etoposide with 45 Gy, followed, in the absence of progression, either by resection or by uninterrupted continuation of radiotherapy to 61 Gy, with consolidation chemotherapy in both arms [21]; the intention-to-treat comparison therefore included events during the shared induction phase. Of 396 eligible patients, 202 were assigned to surgery and 194 to definitive chemoradiotherapy; in the surgical arm, 81% underwent thoracotomy and 71% had a complete resection. Surgery improved local control and progression-free survival (median 12.8 versus 10.5 months; hazard ratio 0.77), but median overall survival was 23.6 versus 22.2 months (hazard ratio 0.87), with no significant difference in the intention-to-treat population. Treatment-related deaths occurred in 8% of the surgical arm and 2% of the non-surgical arm; 14 of the 16 deaths in the surgical arm followed pneumonectomy, which had been performed in 54 patients.

The often-cited lobectomy signal requires caution. In an unplanned exploratory analysis, patients who underwent lobectomy lived longer than patients in the non-surgical arm matched on baseline characteristics (median 33.6 versus 21.7 months), whereas patients who underwent pneumonectomy did not [21]. This was not a randomized lobectomy-specific comparison: the extent of resection was determined after randomization and induction, according to tumor extent, response, and fitness, so the subgroups reflect post-randomization selection that matching could not fully remove, and operative risk differed sharply between lobectomy and pneumonectomy. The analysis supports only the hypothesis that the balance may favor surgery when complete resection is achievable without pneumonectomy; it does not establish that lobectomy is superior to definitive chemoradiotherapy.

The other two trials differed in population and randomization point. EORTC 08941 enrolled patients with pathologically proven stage IIIA-N2 disease judged unresectable at presentation; after induction platinum-based chemotherapy, only responding patients were randomized (332 of 579 eligible patients) to resection or to radiotherapy alone [22]. In the surgical arm, 47% of operated patients underwent pneumonectomy, complete resection was achieved in about half, and postoperative mortality was 4%; median overall survival was 16.4 months after surgery and 17.5 months after radiotherapy. ESPATUE enrolled medically and functionally operable patients with resectable stage IIIA(N2) and selected IIIB disease; after induction chemotherapy and concurrent chemoradiotherapy, patients whose disease remained resectable were randomized (161 of 246 enrolled) to surgery or to a definitive chemoradiotherapy boost [23]. R0 resection was achieved in 81% of patients assigned to surgery. Probably treatment-related deaths after randomization numbered five in the surgery arm (four after lobectomy and one after bilobectomy) and two in the definitive chemoradiotherapy arm [23]. Accrual was stopped early because of slow recruitment and the end of funding, so the comparison was underpowered. Five-year overall survival was 44% with surgery and 40% with definitive chemoradiotherapy, and the 10-year update likewise showed no statistically significant overall-survival difference (29.9% versus 28.3%, respectively; *p* = 0.70) [23,24].

Read together, INT0139, EORTC 08941, and ESPATUE demonstrated superiority for neither platform, but they are not a uniform body of evidence: they randomized at different points, used different non-surgical treatment (radiotherapy alone or concurrent chemoradiotherapy), and differed in pneumonectomy rates and operative mortality. None was designed or powered to establish equivalence or non-inferiority, and ESPATUE closed early; the absence of a significant overall-survival difference means that superiority was not demonstrated, not that the platforms are equivalent. Technical resectability, including apparent suitability for lobectomy, did not by itself establish an overall-survival benefit over the radiotherapy-based platform, nor did that platform establish one over surgery. Because all three trials predate modern staging, surgery, radiotherapy, and systemic therapy, they inform the contemporary question without answering it.

### 3.2. Technical Resectability Defines Eligibility, Not Comparative Platform Suitability

Technical resectability has been applied inconsistently. A 2025 EORTC systematic review found substantial variation in how resectable stage III disease had been defined [25]. The 2026 multisocietal EORTC-led consensus is therefore an important advance. It provides a trial-oriented framework based on stage, single- versus multistation nodal involvement, bulk, invasiveness, modern staging, and review by experienced thoracic surgeons within a multidisciplinary team [15]. It should replace the broad claim that technical resectability cannot be standardized.

Standardization does not resolve the comparative question. A technically removable tumor may still be better treated with chemoradiotherapy if resection requires pneumonectomy or carries a high functional cost. A technically irradiable tumor may be better treated surgically if the target geometry produces excessive cardiac or pulmonary dose. Technical resectability, physiological operability, and protocol-defined irradiability identify patients who can safely receive either platform; comparative benefit can be established only through randomization, not through the label.

This distinction also clarifies the meaning of biological selection. No current genotype, imaging feature, circulating biomarker, or response measure can declare a patient ‘biologically resectable’ or ‘biologically irradiable’; such labels would confuse association within one pathway with prediction of relative benefit between pathways. A factor becomes a platform-selection biomarker only if it modifies the comparative treatment effect, ideally demonstrated by a prespecified treatment-by-factor interaction within randomized data.

### 3.3. Both Platforms Were Established Curative Strategies Before Immunotherapy

Before immunotherapy, both pathways were established curative strategies. Surgery after induction therapy offered durable control in selected patients, particularly when complete resection could be achieved by lobectomy [21,22,23,24]. Concurrent chemoradiotherapy was the established curative framework for bulky, multistation, or fixed N2 disease; RTOG 9410 established concurrent cisplatin-based chemoradiotherapy as superior to sequential treatment, with a long-term survival tail consistent with cure in a subset [26]. Definitive chemoradiotherapy was therefore not merely a fallback for patients unfit for surgery.

### 3.4. Immunotherapy Improved Both Platforms Separately

The evidence supporting immunotherapy within surgery-based treatment is strong (Table 1). CheckMate 816 increased pathological complete response from 2.2% to 24.0% and improved event-free survival with neoadjuvant nivolumab plus chemotherapy; with a median follow-up of 68.4 months, overall survival was also improved (hazard ratio 0.72) [6,7]. KEYNOTE-671 demonstrated event-free and overall-survival benefit with perioperative pembrolizumab [8,9]. CheckMate 77T and AEGEAN improved event-free survival and pathological response with perioperative nivolumab and durvalumab, respectively [10,11], and NADIM II and Neotorch further supported chemoimmunotherapy in resectable stage III disease [12,13,14]. Their control arms were chemotherapy followed by surgery; none used definitive chemoradiotherapy followed by consolidation immunotherapy as the comparator.

The same transformation occurred in the non-surgical pathway. PACIFIC randomized patients with unresectable stage III NSCLC without progression after concurrent platinum-based chemoradiotherapy to durvalumab or placebo; at 5 years, median overall survival was 47.5 versus 29.1 months [3,4,5]. An exploratory stage IIIA-N2 analysis showed preserved benefit, with hazard ratios of 0.46 for progression-free and 0.56 for overall survival [27]. CheckMate 73L, which added nivolumab during chemoradiotherapy, did not improve on PACIFIC-based treatment and increased toxicity [28]; chemoradiotherapy followed by durvalumab therefore remains the non-surgical reference. PACIFIC, in turn, establishes that durvalumab improves the chemoradiotherapy-based pathway; it does not establish that this pathway is equivalent or superior to modern surgery-based treatment in technically resectable patients.

Both pathways have also evolved technically since the historical trials. Contemporary radiotherapy uses more accurate mediastinal and distant staging, motion management, conformal planning, image guidance, and closer attention to cardiac and pulmonary dose [29,30,31,32]. Contemporary thoracic surgery has changed to a comparable degree: video-assisted and robotic approaches, enhanced-recovery pathways, more rigorous patient selection, and bronchovascular sleeve techniques that avoid pneumonectomy in many central tumors have reduced the operative burden represented in older trials. A meta-analysis in N2 disease associated minimally invasive surgery with less blood loss, shorter hospital stay, lower 30-day mortality, and at least comparable oncological outcomes to thoracotomy [20]. Neither evolution establishes superiority over the other pathway; both prevent the historical comparison from being imported unchanged into the present.

**Table 1:** Contemporary randomized trials of immunotherapy within the surgery-based and chemoradiotherapy-based pathways.

Trial; *n*	Population (Resectability Assessment; TNM Edition)	Randomization Point	Systemic Therapy (Experimental vs. Control)	Primary Endpoint(s); Median Follow-Up	N2-Specific Data
*Chemoradiotherapy-based pathway*					
PACIFIC [3,4,5]; *n* = 713	Unresectable stage III (investigator-assessed); TNM 7th	1–42 days after concurrent platinum-based CRT without progression	Durvalumab every 2 weeks for up to 12 months vs. placebo	PFS, OS; 34.2 months (all patients)	Exploratory IIIA-N2: PFS HR 0.46, OS HR 0.56 [27]
*Surgery-based pathway*					
CheckMate 816 [6,7]; *n* = 358	Resectable IB–IIIA (investigator-assessed); TNM 7th	Before neoadjuvant therapy	Nivolumab + platinum doublet ×3 vs. platinum doublet ×3; no adjuvant immunotherapy	EFS, pCR; 68.4 months (OS analysis)	—
KEYNOTE-671 [8,9]; *n* = 797	Resectable II–IIIB (N2) (investigator-assessed); TNM 8th	Before neoadjuvant therapy	Pembrolizumab + cisplatin-based chemotherapy ×4, then adjuvant pembrolizumab ×13 vs. placebo with the same chemotherapy	EFS, OS; 36.6 months	—
AEGEAN [10]; *n* = 802	Resectable II–IIIB (N2) (multidisciplinary assessment; planned pneumonectomy excluded after protocol amendment); TNM 8th	Before neoadjuvant therapy	Durvalumab + platinum-based chemotherapy ×4, then adjuvant durvalumab ×12 vs. placebo with the same chemotherapy	EFS, pCR; 11.7 months (interim analysis)	Exploratory N2 analysis: benefit in single- and multistation N2; multistation estimate less precise [33]
CheckMate 77T [11]; *n* = 461	Resectable IIA–IIIB (N2) (multidisciplinary assessment); TNM 8th	Before neoadjuvant therapy	Nivolumab + platinum-based chemotherapy ×4, then adjuvant nivolumab for 1 year vs. placebo with the same chemotherapy	EFS; 25.4 months	Exploratory nodal-status analysis, including multistation N2 [34]
NADIM II [13]; *n* = 86 (phase 2)	Resectable IIIA–IIIB (T3N2) (multidisciplinary assessment); TNM 8th	Before neoadjuvant therapy	Nivolumab + carboplatin–paclitaxel ×3, then adjuvant nivolumab for 6 months vs. carboplatin–paclitaxel ×3	pCR; 26.1 months	—
Neotorch [14]; *n* = 501 (404 stage III)	Resectable II–III (investigator-assessed); TNM 8th	Before neoadjuvant therapy	Toripalimab + platinum-based chemotherapy ×3, toripalimab + chemotherapy ×1 after surgery, then toripalimab ×13 vs. placebo with the same chemotherapy	EFS, MPR; 18.3 months	—

Note: None of these trials compared the surgery-based and chemoradiotherapy-based pathways with each other. Resectability (or, in PACIFIC, unresectability) was determined by investigators or local multidisciplinary teams; the trials did not use a common definition of eligibility for both pathways [25]. Median follow-up is that of the most recent report cited in each row. The em dash (—) indicates that no dedicated N2 analysis is cited in this review. CRT, chemoradiotherapy; EFS, event-free survival; HR, hazard ratio; MPR, major pathological response; OS, overall survival; pCR, pathological complete response; PFS, progression-free survival; ×*n*, number of cycles.

## 4. Discussion

### 4.1. Better Systemic Control May Make the Local-Platform Question More Important

The historical trials were conducted when distant relapse occurred frequently and survival was shorter. A difference in local treatment could be obscured if many patients developed systemic failure before a local-control advantage had time to influence long-term outcome. The modern systemic backbones raise a plausible, but unproven, possibility: as systemic relapse is delayed and more patients survive longer, durable local control may become more visible and more important [3,4,5,6,7,8,9,10,11,12,13,14,21,22,23,24].

This possibility gives renewed relevance to the randomized local-control gain in INT0139 but does not establish that this advantage persists with contemporary treatment [21]. Equally, it is not established whether consolidation immunotherapy narrows the historical local-control difference after modern chemoradiotherapy [3,4,5,29,30,31,32], whether the difference persists, or whether new patterns of failure emerge. Better systemic treatment may therefore make the local-platform question more important without indicating which platform is better.

### 4.2. The Limits of Cross-Trial Comparison

Population non-comparability is the primary problem. Perioperative trials enrolled patients judged resectable. PACIFIC enrolled patients judged unresectable and randomized them only after chemoradiotherapy had been completed without progression. Even with a better technical definition of resectability, the evidence streams contain different patients selected at different moments.

Endpoint non-comparability is a separate problem. Perioperative event-free survival usually begins before neoadjuvant treatment and can count preoperative progression, inability to undergo surgery, an aborted operation, recurrence, or death [6,8,9,10,11]. It is a pathway endpoint: it measures tumor control and the ability to complete induction, surgery, recovery, and any planned postoperative treatment. PACIFIC progression-free survival begins only after completion of chemoradiotherapy without progression [3,4]. Patients who progressed or could not complete the initial chemoradiotherapy phase are absent from the randomized PACIFIC population.

Definitions of progression-free survival in preoperative therapy vary and produce different estimates and selection biases [35], and surrogate endpoints are not interchangeable with overall survival across settings [36]. A common-baseline event-free-survival definition can compare complete pathways fairly within a randomized trial; the problem lies in comparing differently defined endpoints from separate trials.

Overall survival remains the most definitive survival endpoint, integrating cancer control, salvage, treatment-related mortality, and non-cancer death, but it is not the only relevant outcome. A pathway that produces similar survival with lower functional burden may be preferable even without conventional superiority.

### 4.3. Where Comparative Uncertainty Lies Across the N2 Spectrum

Clearly unresectable disease and disease in medically inoperable patients fall outside the comparative question because only one pathway is feasible. Within the dual-eligible population, practice follows conventions at both ends of the spectrum: low-volume single-station (N2a) disease amenable to lobectomy is commonly treated with surgery, and bulky or multistation (N2b) disease, even when technically resectable, is often treated with chemoradiotherapy [15,18,37]. These conventions reflect reasonable clinical judgment; neither is supported by a modern randomized comparison (Table 2).

Multistation disease, bulky but potentially removable nodes, central tumors, borderline cardiopulmonary reserve, and anticipated pneumonectomy or complex reconstruction define the gray zone, where the trade-offs of each pathway are most visible and the decision is hardest. The gray zone is where comparative uncertainty is most visible and where randomization is most practicable. Outside it, equipoise cannot be assumed, but neither can superiority: it has not been demonstrated that the surgery-based pathway is superior to the CRT-immunotherapy pathway, nor the reverse.

This statement rests on prior randomized evidence rather than on the absence of a trial. INT0139 and ESPATUE enrolled operable patients with resectable disease, and neither showed an overall-survival advantage for either platform [21,23,24]; but neither was confined to single-station disease and both predate modern systemic therapy. Whether either platform confers a net advantage today therefore remains a hypothesis, to be tested through staged extension of a comparative trial rather than assumed in either direction.

Modern perioperative trials have broadened the territory in which surgery is considered. AEGEAN and CheckMate 77T included selected stage IIIB(N2) disease, and exploratory analyses showed activity in N2 disease, including multistation involvement, although multistation estimates were less precise [10,11,33,34]. These data establish activity of perioperative immunotherapy across a broader N2 spectrum, not superiority over CRT-immunotherapy. The converse applies to the stage IIIA-N2 analysis of PACIFIC, which did not enroll patients prospectively confirmed as eligible for both pathways [27].

Two limits on this argument should be explicit. Practical equipoise refers to the willingness of patients, clinicians, and multidisciplinary teams to accept randomization, not to equivalence of the two pathways; and the argument concerns the scope of a research question, not individual clinical obligation, since multidisciplinary decisions appropriately integrate anatomy, reserve, dosimetry, molecular findings, local expertise, and patient preference until comparative evidence exists. Nothing here implies that every patient with low-volume single-station or bulky multistation disease should be randomized; it implies only that neither group should be excluded from comparative research by definition.

### 4.4. Anatomy, Host State, and Biology: Candidate Effect Modifiers, Not Current Selection Rules

Both platforms can cure and both can harm; the comparison is between two multimodality burdens. The penalty is anatomy-dependent, and anticipated extent of resection is among the most consequential variables in multidisciplinary discussion: lobectomy or sleeve resection and pneumonectomy are different propositions. Observational and propensity-matched series report substantially greater non-oncological mortality after pneumonectomy than after lobectomy [16,17]. Conversely, a target geometry that increases cardiac or pulmonary dose shifts risk toward the radiotherapy platform; RTOG 0617 and subsequent analyses linked cardiac dose with worse survival, non-cancer death, and treatment-related immune suppression [29,30,31]. These data do not rank the platforms; they show why anticipated resection extent and predicted dose–volume risk, both assessable before treatment, belong in trial eligibility and stratification.

Host factors may also alter the balance. Age, performance status, pulmonary reserve, cardiac disease, frailty, and recovery after induction therapy can change the cost of surgery more than the cost of chemoradiotherapy, or the reverse. In a comparative trial these variables would need to be measured prospectively rather than treated only as exclusion criteria. Physiological operability is necessary for entry into a surgical arm, but it is not a treatment-selection rule by itself.

Molecular heterogeneity adds another layer. KEAP1/NFE2L2 alterations are associated with oxidative-stress adaptation and clinical signals of radioresistance, whereas STK11/LKB1 alterations are associated with immune-cold biology and adverse outcomes in immunotherapy-treated NSCLC, although their treatment-predictive specificity remains unsettled [38,39,40,41]. These findings do not justify assigning patients with KEAP1-mutant tumors to surgery or selecting chemoradiotherapy by STK11 status. Prognostic adversity within one platform is not evidence of superiority of the alternative platform, and the same alteration may compromise local and systemic components of both pathways.

The anatomical and molecular penalties may stack, and they may point in opposite directions. A patient whose tumor requires pneumonectomy and carries a KEAP1/NFE2L2 alteration illustrates the problem: anatomy loads non-oncological risk onto surgery, while the molecular state raises concern about the chemoradiotherapy-based pathway [16,17,38,39]. Neither signal is sufficient to choose a platform because neither has been shown to modify the comparative treatment effect. Compound cases are precisely where feasibility, intuition, and single-platform associations are least reliable.

Oncogene-addicted disease does not fall outside the comparative question; it changes the systemic backbone. In EGFR-mutant disease, adjuvant osimertinib is established after resection (ADAURA); NeoADAURA demonstrated improved major pathological response with neoadjuvant osimertinib, and LAURA established a progression-free survival benefit after definitive chemoradiotherapy [42,43,44]. LAURA, like PACIFIC, enrolled patients whose disease was judged unresectable without a standardized definition of technical resectability [15,25]. Given the variability with which that label has been applied [25,45], some of these patients might now be considered resectable; for them the appropriate pathway is already uncertain, which is the comparative question in its EGFR-mutant form. InDuRanS excludes both EGFR-mutant and ALK-positive disease [2]. One design option, offered as a hypothesis rather than an established standard, would instead include EGFR-mutant disease as a prespecified stratum with osimertinib in both arms, not powered for a separate confirmatory comparison. For drivers with targeted therapy established in only one pathway (currently ALK, with alectinib established after resection [46] but no equivalent after chemoradiotherapy), the systemic therapies compared would be unequal; deferring patients with these drivers until equivalent standards exist in both pathways is likewise a proposal, not a settled rule.

Biology also changes during treatment. Circulating tumor DNA (ctDNA) after chemoradiotherapy can identify molecular residual disease and recurrence risk and may distinguish post-treatment trajectories [47,48]. Perioperative ctDNA is also of interest. These observations are prognostically important, but they do not establish a rule for choosing surgery over radiotherapy or the reverse. Baseline ctDNA could be tested as a prespecified treatment-effect modifier rather than used as an unvalidated eligibility gate; on-treatment ctDNA, measured at different times after different treatments in the two arms, would be interpreted within each arm.

### 4.5. Clinical Selection Is Moving Faster than Comparative Evidence

Clinical allocation is proceeding more confidently than comparative evidence permits. A review contrasting perioperative chemoimmunotherapy with chemoradiotherapy emphasizes the heterogeneity that prevents direct comparison [49], and expert commentary describes a preference-sensitive population when both approaches are feasible [37,50]. The 2026 EORTC-led consensus standardizes technical resectability for trial inclusion [15], and the 2026 AATS consensus provides practical guidance for N2-positive disease [18]; neither establishes that the pathway judged feasible produces greater net benefit than the alternative.

The 2026 international multidisciplinary Delphi consensus illustrates the same limit [51]. Consensus was reached on induction chemoimmunotherapy as a valid option in medically operable, resectable multistation N2 disease, whereas no consensus was reached for bulky N2 disease or for a standardized approach to persistent N2 disease after neoadjuvant treatment [51]. This map is clinically informative, but it records what experts are prepared to do rather than which pathway produces greater net benefit; acceptance of either pathway as a valid option does not show that it should displace the other.

Non-randomized comparisons cannot close this gap. A national database analysis reported a survival signal favoring induction chemoimmunotherapy followed by surgery over CRT-immunotherapy, which its authors treated as hypothesis-generating [52]; in another analysis, an apparent survival advantage for surgery was no longer significant after propensity matching [53]. Matching balances measured variables but cannot recreate the balance produced by randomization. Contemporary arguments for a central surgical role reach the same boundary: the task is not to defend either pathway universally, but to determine which patients derive greater benefit from each [19].

### 4.6. Emerging Studies Ask Different Versions of the Comparative Question

Several prospective studies are beginning to integrate the surgical and radiation platforms. INCREASE evaluated neoadjuvant chemoradiotherapy with ipilimumab and nivolumab before surgery in resectable and borderline-resectable NSCLC, while SQUAT evaluated neoadjuvant concurrent chemo-immuno-radiation followed by surgery and adjuvant immunotherapy [54,55]. These trimodality studies show that the platforms can be biologically and technically integrated. They do not establish whether surgery should replace definitive chemoradiotherapy after modern systemic treatment.

InDuRanS is more directly relevant. In this ongoing randomized phase II trial, patients with resectable or borderline-resectable stage IIB–IIIB (N2) disease under TNM9, including T1N2a, judged suitable for both local approaches, receive three cycles of durvalumab with platinum-based chemotherapy; those whose disease remains resectable are then randomized to surgery or definitive chemoradiotherapy, followed in both arms by consolidation durvalumab [2]. This design compares the same post-induction patients from the same baseline and therefore removes much of the population and endpoint mismatch; its primary endpoint, two-year event-free survival from randomization in 158 patients, is to be estimated rather than formally tested. Its limitation is equally important: it tests the local platform after shared induction treatment, not the unmodified comparison between a current perioperative surgical pathway and a PACIFIC pathway from diagnosis.

Two rational designs therefore exist, and they answer different questions. A pathway design randomizes at diagnosis between complete contemporary strategies and asks which treatment policy produces the better outcome from the moment of decision; it captures attrition during induction, operative and radiotherapy toxicity, and treatment completion, but requires patients to accept randomization before any response is known. A post-induction design, as in InDuRanS, randomizes after a shared systemic phase and asks which local treatment is better in patients who have responded or remained stable; it may aid recruitment and yield biological information before randomization, although any recruitment advantage remains unproven; however, it excludes patients who progress or deteriorate during induction and therefore does not compare complete pathways. In a post-induction design, mediastinal nodal response, the one dynamic selector already embedded in practice, would be recorded before randomization and analyzed as a candidate effect modifier rather than used to assign the local platform. Historically, the designs that recruited shared an induction phase: INT0139 randomized before a common chemoradiotherapy induction, whereas EORTC 08941 and ESPATUE randomized after induction treatment [21,22,23]. Because the clinical decision is made at diagnosis, the pathway design is proposed here as the primary comparison, with the post-induction design as a pragmatic alternative where recruitment from diagnosis proves infeasible.

### 4.7. Lessons from the PIONEER Feasibility Study

PIONEER provides important feasibility lessons for a definitive trial. Across eight UK centers, 276 patients were screened, 52 were eligible, and 28 were randomized. Uptake of allocated treatment was high, but planned multimodality treatment was completed in only 50% of the surgical arm and 65% of the non-surgical arm. Central review found that 12% of patients labeled unresectable might have been eligible [45]. The investigators concluded that a phase III trial using that design was unlikely to be feasible.

The difficulty may have been structural as well as numerical. Resectability functioned both as an eligibility criterion and, in routine practice, as a treatment-allocation signal, so that randomization was asked to reverse a decision partly made during eligibility assessment. Of 224 excluded patients, lack of physiological fitness accounted for 47%, technical unresectability for 30%, and both for 16%, whereas absence of multidisciplinary equipoise was recorded in only 4% [45]. The population had therefore been narrowed largely by the feasibility labels the trial sought to look beyond. PIONEER was not designed to test this interpretation causally, and it does not show that clearly resectable patients lack comparative uncertainty; it raises the possibility that practical equipoise can be lost when a technical label functions as a treatment recommendation.

The completion rates expose a further point: the intervention is the whole sequence of systemic treatment, local treatment, recovery, and additional therapy, and attrition at any stage is part of the comparative effect. PIONEER therefore does not show that the clinical question is unimportant; it suggests that a trial shaped by local resectability judgments, historical pathways, and pre-existing preferences may face substantial recruitment difficulties.

### 4.8. Implications of PIONEER for a Comparative Trial

**Figure 2:**
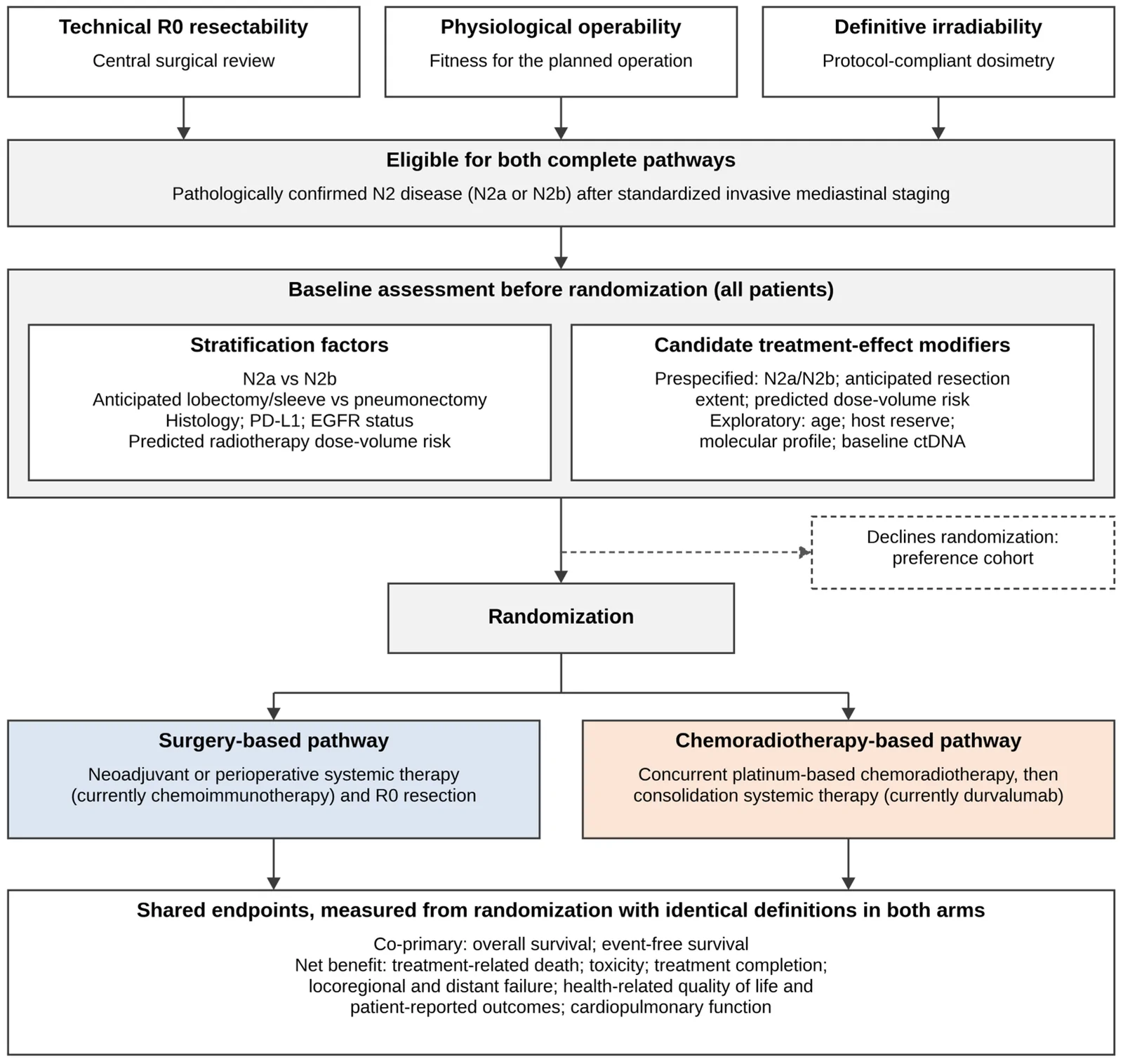
Proposed dual-platform randomized comparison in stage III/N2 NSCLC. Entry criteria (technical R0 resectability, physiological operability, and protocol-compliant irradiability) define the comparative population. Stratification factors and candidate treatment-effect modifiers are baseline variables, assessed before randomization in every patient; they do not assign the pathway, and prespecified modifiers are distinguished from exploratory ones. Patients who decline randomization may be followed in parallel preference cohorts. Randomization from a common baseline compares the complete modern pathways on shared endpoints. In EGFR-mutant disease, osimertinib would replace immunotherapy as the systemic component of both arms (Section 4.4).

The experience of PIONEER does not show that the comparative question cannot be addressed; it identifies the design choices that any comparative trial would have to resolve. The considerations below are not a protocol. First, eligibility would need to be neutral with respect to allocation. Central confirmation of technical R0 resectability, physiological operability, and protocol-compliant irradiability, after standardized invasive nodal staging [56], would define who can receive either pathway; nodal pattern, bulk, and anticipated extent of resection would then stratify the population rather than assign the pathway (Figure 2). PIONEER’s own central review showed that the resectability label was applied inconsistently [45]; central confirmation could reduce that inconsistency, but not the tendency of the label to act as a treatment recommendation, which is what staged accrual is intended to address.

Second, accrual would need to be staged, and feasibility measured rather than assumed: recruitment would begin where comparative uncertainty is most visible (N2b disease, bulky nodes, central tumors, or anticipated pneumonectomy) and extend to N2a disease amenable to lobectomy according to prespecified criteria, with parallel preference cohorts for patients who decline randomization. Third, the arms would be defined as platforms rather than drug regimens, with systemic therapy following approved standards as they evolve (including osimertinib in a prespecified EGFR-mutant stratum), so that the comparison does not become outdated during accrual, as occurred when immunotherapy entered PIONEER by amendment [45]; variation between centers in systemic sequencing and in radiotherapy technique is part of the platforms being compared. Such a trial would be open-label, with blinded central adjudication of event-free survival and locoregional failure.

Fourth, the randomization point would need to be explicit: at diagnosis for the primary comparison of complete pathways, and after induction as the pragmatic alternative (Section 4.6). Common-baseline event-free survival and overall survival, with treatment-related mortality, toxicity, treatment completion, patient-reported outcomes, and cardiopulmonary function, would define net benefit (Table 3); pathological response, measurable only after resection, could describe the surgical arm but could not serve as a comparative endpoint. Finally, a few prespecified treatment-effect modifiers would be tested hierarchically, all defined before randomization and recorded in both arms: N2a versus N2b, anticipated extent of resection judged by central surgical review, and radiotherapy dose–volume risk from a pre-randomization planning assessment. Confirmatory conclusions would require adequate statistical power, and subgroups could not be defined by the operation actually performed or the dose actually delivered.

The statistical principles follow from the same logic and would be specified in a protocol: comparison by assigned pathway in all randomized patients (a treatment-policy estimand), event-free survival measured from randomization with identical definitions in both arms, and control of multiplicity across the two primary endpoints. Overall survival would remain co-primary because INT0139 improved progression-free survival without a significant overall-survival difference [21].

Sample size would be defined at protocol level. The largest contemporary phase III trials in stage II–III disease have enrolled approximately 700–800 patients (Table 1), but none required randomization between surgery-based and chemoradiotherapy-based treatment, a key recruitment challenge identified by PIONEER [45]; InDuRanS, which does, had randomized two patients by August 2026 [2]. Feasibility on that scale would therefore have to be demonstrated prospectively, for example through an internal pilot phase, and would require collaboration among established academic cooperative groups. Restricted or post-induction trials would answer narrower but still important questions.

**Table 2:** Established evidence and remaining uncertainties in stage III/N2 NSCLC.

Domain	Established by Current Evidence	Not Established
Historical local therapy	Surgery improved local control and, in INT0139, PFS; neither platform showed a significant OS advantage in INT0139, EORTC 08941, or ESPATUE, which differed in population, randomization point, and non-surgical treatment [21,22,23,24].	That either platform is superior to the other in N2 disease, or that the two are equivalent; none of these trials was designed to test equivalence.
Low-volume single-station (N2a) disease	Commonly considered technically resectable and frequently treated within a surgery-based pathway [15,18,37].	That lobectomy-based treatment plus modern systemic therapy provides greater net benefit than modern CRT-immunotherapy in a patient eligible for both.
Bulky or multistation (N2b) disease	Commonly treated with CRT-immunotherapy; perioperative immunotherapy is active in selected multistation disease [33,34].	That either pathway provides greater net benefit in this subgroup, or that this subgroup is the only valid population for comparison.
Perioperative immunotherapy	Improves outcomes compared with chemotherapy–surgery controls [6,7,8,9,10,11,12,13,14].	That surgery with modern perioperative systemic therapy is superior to modern CRT-immunotherapy.
CRT-immunotherapy	Durvalumab after definitive concurrent CRT improves PFS and OS compared with CRT alone [3,4,5].	That CRT-immunotherapy is equivalent or superior to modern surgery-based treatment in technically resectable patients.
Technical eligibility	The 2026 technical-resectability consensus can standardize resectability for trials [15,25].	That technical resectability, operability, or irradiability establishes comparative oncological benefit.
Expert consensus	Technical and multidisciplinary consensus can standardize eligibility, describe acceptable practice, and map where agreement is absent [15,18,51].	That expert agreement establishes superiority of one complete pathway over the other.
Endpoints	EFS is valid within a randomized pathway trial when measured from a common baseline; OS is definitive for survival benefit [35,36].	That perioperative EFS and PACIFIC PFS can be compared across trials.
Biomarkers	STK11, KEAP1, ctDNA, and oncogenic drivers are prognostic or treatment-relevant in specific settings [38,39,40,41,42,43,44,46,47,48].	That any current biomarker modifies the comparative surgery-versus-CRT effect sufficiently to serve as a validated platform selector.
Modern technique	Surgical and radiotherapy techniques have evolved and may reduce historical treatment burden [20,29,30,31,32].	That technical modernization has established superiority of either contemporary pathway.

**Table 3:** Design considerations for a contemporary comparative trial (proposals derived from this review, not established standards).

Design Element	Consideration
Population	Pathologically confirmed stage III/N2 disease (N2a or N2b, TNM 9th), including the T1N2a subset now staged IIB, with central confirmation of technical R0 resectability, physiological operability, and protocol-compliant irradiability. Proposed: EGFR-mutant disease as a prespecified stratum with osimertinib in both arms; drivers with targeted therapy established in only one pathway (currently ALK) deferred until equivalent standards exist in both pathways.
Staged accrual	Initial accrual where uncertainty is most visible (N2b, bulky, central, or pneumonectomy-requiring disease), with prespecified feasibility criteria for extension to N2a disease amenable to lobectomy.
Randomization	Open-label. Primary: at diagnosis (complete-pathway comparison). Alternative: after a common induction phase (post-induction local-platform comparison).
Treatment arms	Defined as platforms: systemic therapy follows approved standards in each arm as they evolve (including osimertinib in the EGFR-mutant stratum).
Stratification	Baseline variables assessed in every patient before randomization: N2a versus N2b; anticipated lobectomy/sleeve versus pneumonectomy (central surgical review); histology; PD-L1; EGFR status; predicted radiotherapy dose–volume risk (pre-randomization planning assessment). These factors do not assign the pathway.
Principal endpoints	Common-baseline EFS and OS (co-primary), with identical definitions in both arms; comparison by assigned pathway (treatment-policy estimand).
Net-benefit endpoints	Treatment-related death, non-cancer mortality, grade 3–5 toxicity, local and distant failure, treatment completion, HRQoL and other patient-reported outcomes, pulmonary and cardiac function, treatment preference.
Recruitment	Internal pilot phase with prespecified feasibility criteria; preference cohorts for patients declining randomization; collaboration among established academic cooperative groups.
Quality assurance	Standardized invasive nodal staging (ESTS); central surgical and radiotherapy review; prospective dosimetry; blinded central adjudication of EFS and locoregional failure.
Translational plan	Prespecified interactions (baseline variables recorded in both arms): N2a/N2b, anticipated extent of resection, predicted dose–volume risk. Exploratory baseline variables: age, host reserve, molecular profile, baseline ctDNA. On-treatment measures (ctDNA dynamics; pCR/MPR in the surgical arm) are descriptive within arms, not comparative modifiers or endpoints. Explicit separation of prognostic from predictive effects.

## 5. Conclusions

Immune checkpoint inhibition has improved both curative pathways in stage III/N2 NSCLC without comparing them with each other in dual-eligible patients. Historical randomization showed better local control with surgery but no overall-survival advantage for either pathway, without establishing that the two are equivalent. Better systemic control may make the choice of local platform more important, but it does not show which platform is better.

Technical resectability, physiological operability, and irradiability define eligibility, not comparative benefit. A strong preference for lobectomy in favorable N2a disease is not randomized evidence of superiority, nor is the conventional preference for chemoradiotherapy in multistation disease. Until comparative evidence exists, multidisciplinary judgment that integrates anatomy, physiology, dosimetry, biology, and patient preference remains the appropriate basis for treatment decisions.

A staged randomized comparison of the complete modern pathways, beginning where uncertainty is most visible, extending across the dual-eligible N2 spectrum according to prespecified criteria, and testing prespecified baseline effect modifiers hierarchically, is proposed as one route to that evidence. Feasibility defines what can be done; comparative evidence is required to determine what should be done.

## Data Availability

No new data were created or analyzed in this study. Data sharing is not applicable to this article.
